# A review of scoring systems for ocular involvement in chronic cutaneous bullous diseases

**DOI:** 10.1186/s13023-018-0823-5

**Published:** 2018-05-22

**Authors:** Brendon W. H. Lee, Jeremy C. K. Tan, Melissa Radjenovic, Minas T. Coroneo, Dedee F. Murrell

**Affiliations:** 1 Faculty of Medicine, University of New South Wales, Sydney, 2052 Australia; 2 Department of Ophthalmology, Prince of Wales Hospital, Sydney, 2031 Australia; 3 Department of Dermatology, Ground Floor, James Laws House, St George Hospital, Kogarah, Sydney, NSW 2217 Australia; 4 Ophthalmic Surgeons, Sydney, 2031 Australia

**Keywords:** Epidermolysis bullosa, Autoimmune blistering diseases, Disease severity, Scoring tools/systems, Ocular surface disease, Oculocutaneous, Cicatrising conjunctivitis

## Abstract

**Background:**

Epidermolysis bullosa (EB) and autoimmune blistering diseases (AIBD) describe a group of rare chronic dermatoses characterized by cutaneous fragility and blistering. Although uncommon, significant ocular surface disease (OSD) may occur in both and require ophthalmological assessment. Disease scoring systems have a critical role in providing objective and accurate assessment of disease severity. The objectives of this report were, firstly, to document the prevalence and severity of ocular involvement in EB/AIBD. Secondly, to review and evaluate existing ocular and systemic scoring systems for EB/AIBD. Finally, to identify areas where further development of ocular specific tools in EB/AIBD could be pursued.

**Methods:**

A literature search was performed in October 2017 utilising Medline, Embase, and Scopus databases. The results were restricted by date of publication, between 01.01.1950 and 31.10.2017. The reference lists of these articles were then reviewed for additional relevant publications. Articles of all languages were included if an English translation was available. Articles were excluded if they were duplicates, had no reference to ocular involvement in EB/AIBD or described ocular involvement in other diseases.

**Results:**

Descriptions of ocular involvement in EB/AIBD were identified in 88 peer-reviewed journal articles. Findings reported include but are not limited to: cicatrising conjunctivitis, meibomian gland dysfunction, dry eye disease, trichiasis, symblepharon, fornix fibrosis, keratopathy, ectropion/entropion, ankyloblepharon, corneal ulceration, visual impairment and blindness. Although scoring systems exist for assessment of OSD in mucous membrane pemphigoid, no such tools exist for the other AIBD subtypes or for EB. Several systemic scoring systems exist in the dermatological literature that are efficacious in grading overall EB/AIBD severity, but have limited inclusion of ocular features. To the best of our knowledge, there is no recognised or validated scoring systems which comprehensively stages or grades the spectrum of ocular manifestations in EB/AIBD.

**Conclusions:**

There are a range of ocular complications documented in EB and AIBD. Development of a comprehensive ocular scoring system for EB/AIBD which incorporates the delineation between ‘activity’ and ‘damage’ would facilitate more objective patient assessment, improved longitudinal monitoring, comparison of intervention outcomes, and provide commonality for discussion of these patients due to the multidisciplinary nature of their care.

## Background

Chronic cutaneous bullous diseases, including inherited epidermolysis bullosa (EB) and autoimmune blistering diseases (AIBD) cause significant morbidity and mortality [1, 2]. They predominantly manifest with cutaneous signs, but can also involve all mucous membranes of the body, including those in the ocular, oral, and genitourinary areas. The range and severity of ocular involvement is thought to be due to the various biochemical and ultrastructural similarities common to the skin, conjunctiva, and cornea, which are both embryonically derived from the surface ectoderm [3, 4]. Disease scoring systems have a critical role in providing objective and accurate assessment of disease severity. A clear framework and validation is important to ensure the reliability and accuracy of these tools. Further, severity scores from such tools can be utilised to guide treatment decisions and evaluate outcomes. Both EB/AIBD have been documented to cause severe ocular complications [3, 5–8]. Thus, careful ophthalmological assessment, ideally with the aid of a validated scoring system, should be an essential part of the multidisciplinary management of these patients.

The purpose of this report is to document the prevalence and severity of ocular involvement in EB/AIBD. Further, this report will review and evaluate existing scoring systems for ocular involvement in EB/AIBD. Finally, this report will discuss the trends in other ophthalmic and dermatological scoring systems, and their potential use in identifying and furthering the development of EB/AIBD ocular specific tools.

## Methods

The literature search was performed in October 2017 using three online databases: Medline, Embase, and Scopus. The following search terms were used: [‘epidermolysis bullosa’] or [‘autoimmune blistering diseases’] in combinations with [‘review’, ‘ocular involvement’, ‘eye’, ‘clinical ocular disease assessment tool’, ‘eyelid’ ‘conjunctiva’, ‘cornea’, ‘disease severity’, ‘disease scoring’, ‘eye disease’, ‘vision’, ‘fornix’, ‘grading’, ‘progression’, ‘staging system’, ‘scoring system’]. The results were restricted to articles published from 01.01.1950 to 31.10.2017. The reference lists of these articles were also reviewed for additional relevant publications. Articles of all languages were included, if an English translation was available, and any duplicates were removed. An abstract screen of all articles was then performed by two authors (BWHL, MR). Articles were excluded if they discussed EB/AIBD with no reference to ocular involvement or discussed ocular involvement in other diseases with no reference to EB or AIBD. A total of 88 peer-reviewed journal articles describing ocular involvement in EB and/or AIBD were identified. Full copies of the relevant papers were then obtained and reviewed.

### Ocular involvement in EB

All EB types are characterized by mechanical fragility and blistering, but each major type can be differentiated by the level of skin cleavage. These types can be further distinguished by phenotypic characteristics, mode of inheritance, targeted proteins, distinctive patterns of immunofluorescence antigen mapping or on transmission electron microscopy, and mutational analysis [9].

In 2014, the Fifth International Consensus Meeting on EB Diagnosis and Classification established an ‘onion skin’ approach to subclassifying the extensive list of more than 30 phenotypic subtypes [9]. There are four major types of inherited EB: EB simplex (EBS), Junctional EB (JEB), Dystrophic EB (DEB), and Kindler syndrome (KS). EBS encompasses all subtypes that are confined to the epidermis. EBS can be further subtyped into suprabasal EBS, which targets proteins: transglutaminase 5, plakophilin 1, desmoplakin, and plakoglobin, or basal EBS, which involves keratins 5 and 14, exophilin 5, and bullous pemphigoid antigen 1 (BP230). JEB subtypes develop within the mid portion/junction of the skin basement membrane zone (BMZ), otherwise known as the lamina lucida. Affected proteins in JEB include laminin-322, bullous pemphigoid antigen 2 (BP180), and α6β4 integrin subunits. DEB is split into dominant DEB (DDEB) and recessive DEB (RDEB) subtypes, both of which collectively target collagen VII and occur within the uppermost dermis, beneath the lamina densa of the skin BMZ. Finally, Kindler syndrome presents with a mixed pattern, targeting the kindlin-1 protein, and can uniquely arise in multiple levels within or beneath the skin BMZ.

**Fig. 1:**
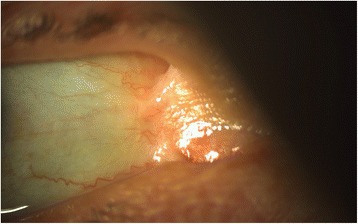
Symblepharon in a patient with JEB seen on slit lamp examination. Symblepharon are adhesions, partial or complete, of the palpebral conjunctiva of the eyelid to the bulbar conjunctiva of the eyeball

The first report of ocular involvement in EB was in 1904 [10]. Since then, case reports [11–20] and case series [21–25] have reported the ocular manifestations occurring in various EB subtypes. Findings can arise as early as 1 month of age and predominantly involve the anterior segment of the eye [26]. These findings may be symptomatic or asymptomatic and present acutely or chronically [27]. Collectively termed Ocular Surface Disease (OSD), findings can include but are not limited to: blepharitis [23], lacrimal duct obstruction [24, 25], blepharoconjunctivitis [19, 21, 23], symblepharon (Fig. 1) [15–17, 21], ankyloblepharon [24], ectropion/entropion [24, 25, 28], corneal abrasions/erosions [14, 17, 19–21, 23–25, 28], pannus formation [22, 24, 25], keratopathy [17, 25, 28, 29], and scarring [17, 22–25, 27]. Progressive visual impairment resulting in blindness has also been reported [27, 30, 31].

One of the largest case series of ocular involvement in EB was by Tong et al. who examined the ophthalmological records of 181 consecutive patients at Great Ormond Street Children’s Hospital (United Kingdom) from 1980 to 1996 [25]. Reported frequencies of ocular complications were 12, 40, 4, and 51% in EBS, JEB, DDEB, and RDEB respectively. These findings were comparable to case series completed by Lin et al. at Rockefeller University (New York) from 1986 to 1993 on 204 EB patients and by Gans at Washington University (New York) from 1979 to 1986 on 78 EB patients [23, 24]. Two sequential case series from St Thomas’ Hospital (London) included the first reports of limbal broadening in DEB patients [21, 22]. Limbal broadening has been described as clouding of the corneal periphery adjacent to the limbus [32]. However, the term has rarely been used in the literature, and no established definition currently exists. KS was added under the EB umbrella in 2008 [33]. A recent Palestinian study on the largest affected family (*n* = 18) revealed findings of ectropion and keratoconjunctivitis in all patients, early development of symblepharon in 17 cases, and blindness in one [31].

These case series were then followed by a landmark retrospective account by Fine et al. who examined 3280 consecutively enrolled patients from the National EB Registry (NEBR), a National Institutes of Health database funded from 1986 to 2002 [27]. The NEBR had 16 years of methodical follow-up and is the only significant longitudinal data on ocular involvement in EB to date [34]. A concise representation of their findings can be seen in Table 1. Fine et al. noted that the frequency of ocular involvement mirrored severity of skin disease. In particular, a high occurrence of corneal erosions/blisters were noted in RDEB (74.10, 32.45, 35.29%) and JEB (47.50, 25.26%) subtypes. Corneal scarring, symblepharon, and ectropions were also reported at elevated frequencies in these subtypes. In addition, the authors noted the dramatically increased cumulative risks of nonscarring and scarring corneal lesions in a JEB subtype (age 5; 83.18, 27.08% and age 25; 83.18, 72.22%). In contrast, EBS and DDEB subtypes had lower rates of occurrence of each ocular complication, except for visual impairment, which was prevalent across all EB types.

**Table 1:** Frequency of ocular findings in the National EB Registry study population by EB subtype (%)

	EB Simplex				Junctional EB		Dystrophic EB			
Manifestation	Localised (*N* = 1092)	G-S (*N* = 113)	G-I (*N*-96)	Ogna (*N* = 379)	G-S (*N* = 40)	G-I (*N* = 190)	DDEB G (*N* = 424	RDEB G-S (*N* = 139)	RDEB G-I (*N* = 265)	RDEB Inversa (*N* = 17)
Corneal erosions/blisters	0.92	6.19	3.13	2.64	47.50	25.26	2.12	74.10	32.45	35.29
Corneal scarring	0.27	0.00	3.16	0.53	26.83	13.37	0.95	50.00	16.92	29.41
Symblepharon	0.00	0.00	0.00	0.00	4.76	2.11	0.00	10.07	1.89	11.76
Blepharitis	0.37	0.88	2.08	0.26	7.14	6.32	0.71	17.52	6.46	17.65
Ectropion	0.00	0.00	0.00	0.00	14.29	2.11	0.00	7.19	1.90	0.00
Lacrimal duct obstruction	1.19	2.65	1.04	1.85	2.38	4.23	1.65	5.80	5.30	11.76
Impaired vision	13.17	13.27	15.63	16.14	16.67	13.68	17.18	38.13	21.89	41.18
Blindness	0.82	1.77	0.00	0.53	0.00	1.58	0.94	6.47	1.14	0.00

Abbreviations: *G-S* = generalised-severe; *G-I* = generalised-intermediate; *G* = generalised

Source: Fine JD, Johnson LB, Weiner M, Stein A, Cash S, Deleoz J, et al. Eye involvement in inherited epidermolysis bullosa: experience of the National Epidermolysis Bullosa Registry. Am J Ophthalmol. 2004;138:254–262. (Permission for reuse obtained under RightsLink)

Since the NEBR study, there have been minimal large-scale studies on EB. An examination of 55 children with EB reported 38% had reduced corrected distance visual acuity (≤6/12) in at least one eye and 29% had refractive error [35]. However, this study lacked longitudinal follow-up, therefore it is unclear whether this is due to permanent scarring or ongoing disease. Other studies have shown that visual acuity can transiently decline during corneal insults like erosions [36]. Another recent prospective study examined the meibomian gland dysfunction in 105 children with EB using a recognised classification system [37] and reported that 87.62% exhibited one or more features of dysfunction [38]. Previous reports of EB did not have dedicated meibomian gland assessment and were limited to measuring the absence or presence of blepharitis, which varied from 0.37–17.65% depending on the EB subtype [23, 39].

### Ocular involvement in AIBD

Ocular involvement across AIBD varies in frequency and severity. The main pathological process involves autoimmune-induced conjunctival inflammation with consequent cicatrising outcomes [40, 41].

### Mucous membrane pemphigoid (MMP)

MMP is a group of AIBDs that affects one or more mucous membranes. It is defined by an immune mediated attack of the basement membrane of mucosal surfaces and resulting infiltration of activated inflammatory cells [42]. The target antigens of these autoantibodies are similar to the proteins deficient in EB, namely, laminin-322, laminin-311, laminin-γ1, α6β4 integrin subunits, collagen VII, BP230, and BP180 [43, 44]. MMP with ocular involvement (OcMMP), previously known as ocular cicatricial pemphigoid, is a sight-threatening disease that presents insidiously as chronic conjunctivitis [45]. OcMMP is rarely unilateral and may occur in isolation or in association with other mucous membranes or the skin [46, 47]. OcMMP has been estimated to occur in 64–89% of MMP patients [48–51], usually between the ages of 30–90, with predominance in females [48, 52, 53]. A study of 36 MMP patients concluded that OcMMP is significantly more severe and progressive in younger patients, despite immunosuppression [54]. Patients often report photophobia, tearing, burning, mucous discharge or foreign body sensations, while clinicians can observe erythema or signs of scarring [55]. Disease progression is typically characterized by cicatrising conjunctivitis with subepithelial fibrosis that eventuates in symblepharon formation and fornix foreshortening, typically affecting the inferior fornix first [48, 56, 57]. Medial canthal scarring, with loss of plica and caruncle, has also been touted as a common early sign [44]. Reports of the scarring process destroying goblet cells, lacrimal gland ductules, and meibomian gland orifices leading to dry eye disease (DED) have also been documented [8]. Advanced disease consists of lagophthalmos, trichiasis, ectropions, entropions, ankyloblepharons, and corneal ulceration [7]. Signs of cicatrising ocular disease without symptoms has been seen in 9 MMP patients, demonstrating OcMMP’s potential to be asymptomatic [58]. If not diagnosed or treated early, progression to severe OSD causing vision loss has been reported to occur in 33% of patients [41, 46].

### Pemphigus

**Fig. 2:**
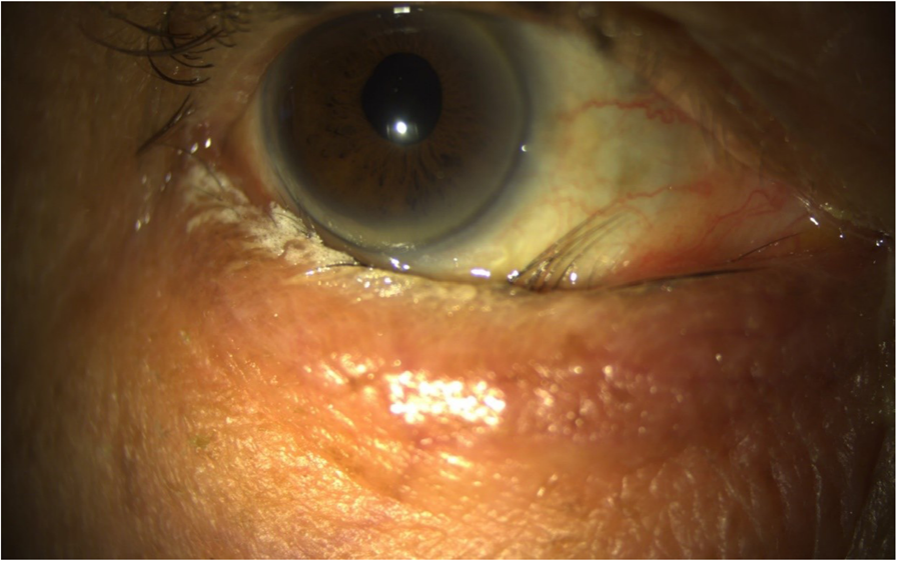
Entropion causing trichiasis in a patient with pemphigus vulgaris seen on slit lamp examination. Entropion is the inward turning of the eyelid, and more conmmonly affects the lower eyelid. This can be complicated by trichiasis, where the eyelids are misdirected inwards towards the eye. Trichiasis can potentially cause severe ocular irritation due to constant contact with the palpebral conjunctiva, bulbar conjunctiva or the cornea

Pemphigus vulgaris (PV), pemphigus foliaceus (PF), and paraneoplastic pemphigus (PNP) describe a group of AIBDs that are categorized by IgG autoantibodies that target intraepidermal desmosomal adhesion proteins, particularly desmoglein 3 and desmoglein 1. This process is characterized histologically by intraepithelial cleavage and separation of epidermal cells, ultimately leading to acantholysis of keratinocytes and the formation of flaccid blisters [59]. Ocular involvement in PV was previously thought to be rare, however more recent literature on Iranian and Spanish PV cohorts reported incidences of 16.5% (17/103) and 14.3% (24/167), contrasting this assertion [60, 61]. Common presentations include irritation, tearing or foreign body sensation [62]. Findings of bilateral conjunctivitis, lid margin ulceration, fornix foreshortening, symblepharon, ankyloblepharon, and entropion (Fig. 2) have all been reported in the literature, indicating the potential for cicatricial changes [63–65]. Limbal broadening and clinical evidence indicating DED have also been reported [5]. It has been postulated that the ocular manifestations may herald the onset of cutaneous involvement [40] – however, the appearance is often unpredictable and may not correlate with severity [65]. Ocular PF is very rare and usually limited to the eyelid skin without compromise of the conjunctiva. Main features include dysmorphic eyelids, dry eyelid skin, madarosis, and subepithelial fibrosis [66]. In contrast, ocular involvement has been reported to occur in 66–72% of PNP patients [7]. Retinal damage, uveitis, blepharospasm, and progression to cicatrising disease have been described. [40, 67] However, specific data is scarce and only limited reports exist due to the rarity of the condition [68–70].

### Linear immunoglobulin-A bullous dermatosis (LABD)

LABD is characterized by blistering from IgA autoantibodies targeting the dermo-epidermal adhesion complex, with BP180 being the main antigen. Ocular involvement occurs in 50–60% of patients with LABD who have DED or report symptoms such as foreign body sensation, burning, and mucous discharge [71–73]. Examination can reveal conjunctival scarring and subconjunctival fibrosis with secondary corneal clouding that leads to vision impairment [74]. Progression to entropion, trichiasis, corneal opacification, and blindness have also been reported [74, 75]. It is important to note that ocular findings may be indistinguishable from OcMMP [8]. There has been one case of LABD with exclusive eye involvement [76].

### Epidermolysis bullosa acquisita (EBA)

EBA, not to be confused with inherited EB, is a disorder of IgG autoantibodies to collagen VII, the same protein affected in DEB. Although ocular involvement has been documented in the literature, the incidence or prevalence has not been recorded [77–81]. Reported manifestations include keratitis, corneal vesiculation, and frequent symblepharon formation – however, vision loss is rare [40, 80]. Ulcerative keratitis in the absence of scarring has also been noted, suggesting corneal inflammation could be a direct manifestation of EBA [57]. In addition, a subtype ‘IgA-EBA’, with predominant IgA autoantibodies, is associated with severe ocular disease that is potentially refractory to treatment [78, 82–84]. Blindness has been reported in this subtype [77, 78, 85].

### Other

Bullous pemphigoid (BP) is the most common AIBD, affecting proteins BP180 and BP230 – however, extra-cutaneous membranes are only occasionally involved in BP and there is limited literature on ocular involvement [5, 86, 87]. Conjunctival scarring has been reported as occurring infrequently [50]. More recently, limbal broadening and DED were novel signs seen in an Australian cohort of BP patients [5]. Documentation of ocular manifestations in anti-p200 pemphigoid, lichen pemphigoides, and pemphigus gestationis have been limited due to their rarity [43]. Lastly, ocular involvement in dermatitis herpetiformis is rare, confined to peri-orbital areas, and rarely results in cicatrising changes [86].

### Ocular involvement scoring systems in EB

There is considerable variation in the types of ocular complications previously reported in EB patients (Table 2). To the best of our knowledge, no validated ocular scoring system in EB exists.

**Table 2:** Ocular complications of EB reported in previous case series

Gans (1988)	Lin et al. (1994)	Tong et al. (1999)	Fine et al. (2004)
Corneal erosions	Corneal abrasion	Corneal abrasion, pannus or scar	Corneal erosions or blisters
Corneal scar	Corneal scar		Corneal scar
–	Pannus		–
Eyelid blister	Eyelid blister	–	–
Blepharitis	–	–	Blepharitis
–	Ectropion	Eyelid ectropion/entropion	Ectropion
–	Symblepharon	Symblepharon	Symblepharon
–	Eyelid scar	–	–
–	Conjunctival blister	Conjunctival blister	–
	–	Punctate keratitis	–
–	–	–	Lacrimal gland obstruction
–	–	–	Impaired vision
–	–	–	Blindness

*Data from:* Gans LA. Eye lesions of epidermolysis bullosa. Clinical features, management, and prognosis. Arch Dermatol. 1988;124:762–764.; Lin AN, Murphy F, Brodie SE, Carter DM. Review of ophthalmic findings in 204 patients with epidermolysis bullosa. Am J Ophthalmol. 1994;118:384–390.; Tong L, Hodgkins PR, Denyer J, Brosnahan D, Harper J, Russell-Eggitt I, et al. The eye in epidermolysis bullosa. Br J Ophthalmol. 1999;83:323–326.; Fine JD, Johnson LB, Weiner M, Stein A, Cash S, Deleoz J, et al. Eye involvement in inherited epidermolysis bullosa: experience of the National Epidermolysis Bullosa Registry. Am J Ophthalmol. 2004;138:254–262

Systemic EB disease scoring systems have, however, been published in the dermatological literature. The earliest tool was developed in Japan, but didn’t assess any element of ocular involvement [88]. This was followed by The Birmingham EB Severity Score in 2009, which aimed to score severity of all EB subtypes by assessing percentage of skin affected. It was the first tool to feature an ocular scoring component, although this was limited (Table 3) [89]. Similarly, the Instrument for Scoring Clinical Outcome of Research for EB, created in 2015, also featured limited ocular scoring (5% of total score) (Table 4) [90]. In contrast, the Epidermolysis Bullosa Disease Activity and Scarring Index (EBDASI), developed in 2014, was the first systemic EB tool to include a subsection scoring ocular involvement with differentiation between disease ‘activity’ and ‘damage’ (Table 5) [91]. Although the EBDASI has an excellent application in the objective assessment of overall EB severity, the ocular scoring component is still far from comprehensive.

**Table 3:** Ocular scoring in the Birmingham Epidermolysis Bullosa Severity Score

Eyes	Score
No problem from EB	0
Occasional soreness	1
Frequent soreness	2
Persistent soreness Early visible external eye disease	3
Between Score 3 and 5	4
Bilateral sight-threatening eye disease	5

*Adapted from:* Moss C, Wong A, Davies P. The Birmingham Epidermolysis Bullosa Severity score: development and validation. Br J Dermatol. 2009;160:1057–1065

**Table 4:** Ocular scoring in the Instrument for Scoring Clinical Outcome of Research for EB

Eye redness	Score	Palpebral closure	Score
Absent	0	Full closure	0
1–2 days/month	1	White to inferior conjunctiva	1
1–2 days/week	2	White to cornea	2
≥3 days/week	3	White to pupil	3

*Adapted from:* Schwieger-Briel A, Chakkittakandiyil A, Lara-Corrales I, Aujla N, Lane AT, Lucky AW, et al. Instrument for scoring clinical outcome of research for epidermolysis bullosa: a consensus-generated clinical research tool. Pediatr Dermatol. 2015;32:41–52

**Table 5:** Ocular scoring in the EB Disease Activity and Scarring Index

Activity		Damage	
Erosions/blisters/erythema/mucosal atrophy/fissures/stenosis	Score	Lesions	Score (0 = absent) (2 = present)
Absent	0	Ectropion	
1 lesion	1	Symblepharon	
2–3 lesions	2	Visible corneal opacity	
> 3 lesions or 2 lesions > 2 cm	5		
Entire area	10		
Total activity score	/10	Total damage score	/6

*Adapted from:* Loh CCH, Kim J, Su JC, Daniel BS, Venugopal SS, Rhodes LM, et al. Development, reliability, and validity of a novel Epidermolysis Bullosa Disease Activity and Scarring Index (EBDASI). J Am Acad Dermatol. 2014;70:89–97

### Ocular involvement scoring systems in AIBD

There has been a range of scoring systems developed to stage ocular cicatricial changes in OcMMP, but almost none in the other AIBDs. The first two clinical systems used to stage OcMMP were described by Foster [48, 49] and Mondino & Brown [53, 92, 93]. While both systems classified OcMMP into one of four distinct stages, Mondino & Brown estimated inferior fornix foreshortening, whereas Foster considered certain clinical features such as symblepharon and fibrosis. In 1992, Tauber et al. cited the insensitivity of these systems individually and proposed a combined format (Table 6) [94].

**Table 6:** Foster (1986), Mondino (1987), and Tauber (1992) systems for mucous membrane pemphigoid with ocular involvement

Foster (1986) and Mondino (1987).		
System		Characteristics
Foster stages	I	Subconjunctival scarring and fibrosis
	II	Fornix foreshortening of any degree
	III	Presence of symblepharon, any degree
	IV	Ankyloblepharon, frozen globe
Mondino stages	I	0–25% loss of inferior fornix depth
	II	25–50% loss of inferior fornix depth
	III	50–75% loss of inferior fornix depth
	IV	75–100% loss of inferior fornix depth
Tauber Staging System (1992).		
I		Subconjunctival scarring and fibrosis
II	a	0–25% loss of inferior fornix depth
	b	25–50% loss of inferior fornix depth
	c	50–75% loss of inferior fornix depth
	d	75–100% loss of inferior fornix depth
III	a	0–25% horizontal involvement of symblephara
	b	25–50% horizontal involvement of symblephara
	c	50–75% horizontal involvement of symblephara
	d	75–100% horizontal involvement of symblephara
	n	Number of symblephara countable
IV		Ankyloblepharon, frozen globe

*Adapted from:* Tauber J, Jabbur N, Foster CS. Improved detection of disease progression in ocular cicatricial pemphigoid. Cornea. 1992;11:446–451

More recently, the use of custom-designed metric rulers to measure fornix depth have been developed, allowing more accurate quantification of the degree of damage [95, 96]. This has allowed measurement of the upper fornix, which had previously been omitted due to difficulty of access. Validation studies establishing normal upper and lower fornix baseline values were completed on South Asian and Caucasian populations [97, 98]. Even so, these scoring systems were limited because they attempted to measure the conjunctival surface area that existed in a curved three-dimensional structure. Efforts to expose the conjunctiva would cause distortion leading to significant variation, which is further compounded by the partially hidden nature of the conjunctiva and fibrosed fornixes that occurs in advanced OcMMP [99].

A new method of measuring the ocular surface was developed in 2004 by Rowsey et al., which estimated the bulbar and tarsal conjunctiva involvement by quantifying the degree of contracture between the lid margin and limbus in three different gaze positions (5-, 6-, and 7-o’clock positions) [100]. The authors noted that the total cumulative measurement in a normal conjunctiva was approximately 45 mm, and stipulated that a decrease in 3 mm indicated disease progression. This was followed by Reeves et al. in 2012, whose method aimed to quantify vertical forniceal depth and horizontal involvement of the inferior conjunctival along the bulbar aspect [99]. While the method proposed by Rowsey et al. avoided distortion of the conjunctiva, it did not directly measure the degree of subepithelial fibrosis. Reeves et al. accounted for this in their system and concluded that completing both methods together would provide the most complete grading [99]. However, this has been contested by more recent literature [97]. Jutley et al. highlighted the lack of intra- and inter-observer reliability or validation. They also argued that increased variability could occur if conjunctival involvement was minimal, which would decrease the chance of detecting early OcMMP [97]. Furthermore, lid laxity, which is prevalent in the OcMMP population, can be problematic by preventing enough stretch in the lid to allow adequate measurement of the globe.

These studies represent considerable advancement in refining the accuracy of OcMMP scoring and staging. However, they are predominantly focussed on grading severity from chronic signs of ‘damage’ over time such as fornix fibrosis, and do not account for the degree of disease ‘activity’, such as concurrent inflammation. Furthermore, these systems rely on the shifts that occur between assessments to infer disease activity and progression.

By contrast, in 1990, Francis et al. created a OcMMP tool that comprised an extensive range of clinical findings that included both ‘damage’ and ‘activity’ parameters, though they didn’t make this distinction [101]. Notable features graded include: conjunctival inflammation, visual acuity, Schirmer’s test, lagophthalmos, symblepharon, fornix shortening/fibrosis, medical canthal keratinisation, trichiasis, corneal scarring, and current infection. Evaluation of the ocular surface using fluorescein and Rose-Bengal staining were also carried out. The authors placed specific emphasis on medial canthal keratinisation and postulated its presence as a reliable diagnostic indicator for OcMMP [102, 103]. Francis et al.’s tool also employed a numerical grading system, which could then be converted to a percentage score (total maximum score of 100%). The authors believed that this method of scoring, in addition to the multitude of clinical aspects recorded, would provide a more quantifiable method of grading OcMMP disease activity, progression, and response to therapy. However, despite the system’s comprehensive design, it was never validated or adopted for widespread use, and only utilised in small retrospective studies.

More recently, Munyangango et al. developed a method for scoring active disease in OcMMP, by dividing each eye into four quadrants and measuring erythema from 1 to 4 (+) for each quadrant [104]. This conjunctival inflammation scoring tool was later adopted into a systemic MMP scoring tool by consensus of an international panel of bullous diseases experts at a MMP conference [105].

Lastly, in 2017, Tepelus et al. piloted the use of in vivo confocal microscopy (IVCM) to gather comprehensive morphological data on changes of the corneal epithelial layers, stroma, endothelium, and presence of inflammatory dendritic cells (DC) in OcMMP [106]. IVCM provides high-resolution images that facilitate ocular surface assessment in a minimally invasive fashion [107, 108]. Their findings were consistent with previous studies using IVCM in OcMMP, but also included several new findings, such as significantly increased density of epithelial DCs [109–111]. Elevated DCs have been demonstrated in patients with DED and could contribute to quantification of disease activity [107, 108].

Like EB, several systemic scoring systems exist for the other AIBD groups, such as the Autoimmune Bullous Skin Disorder Intensity Score [112] and Pemphigus Disease Area Instrument [113, 114]. The former provided no eye assessment, while the latter included a score for ocular ‘activity’ amounting to 3.8% of the total score. Subsequent systems, developed by an international panel of dermatological and bullous disease experts, like the Bullous Pemphigoid Disease Area Index in 2012 [115] and the Mucous Membrane Pemphigoid Disease Area Index in 2015 [105], have recognised the impact of ocular involvement and incorporated more dedicated ocular scoring components.

Though these systems are efficacious at quantifying overall clinical severity of EB/AIBD in patients, their methods of ocular scoring are limited to only a few features. Nevertheless, these studies highlight the importance of ocular involvement in these bullous dermatoses, and as a result, ophthalmological examinations on EB/AIBD patients have become more comprehensive with inclusion of ocular surface and DED evaluations [5, 54, 60, 61]. For example, Tan et al. utilised a system that incorporated the OSD Index [116], a validated 12-item questionnaire developed by the Outcomes Research Group at Allergen Inc. (Irvine, California) to rapidly test subjective impact of DED symptoms, and a data collection sheet that documented 20 distinct ocular lesions segregated into eyelid, cornea, and conjunctiva [5]. In addition, conjunctival inflammation and DED were objectively graded using established methods [5]. Even with these advancements, the development of a specific ocular scoring tool for AIBD is still lacking. To the best of our knowledge, a comprehensive validated system that grades the severity and range of ocular complications still does not exist.

### The future: differentiating activity from damage

A search of the literature for analogous conditions that can cause OSD, like Stevens-Johnson syndrome (SJS), is useful to inform development of a scoring system for EB/AIBD. Sotozono et al. aimed to score the severity of chronic ocular manifestations in SJS by creating an itemised form of 13 complications segregated into either cornea, conjunctival, or eyelid [117]. Gregory concluded that the use of a visual acuity assessment, dry eye questionnaire by Gulati et al. [118], DED measurement guidelines by the International Dry Eye Workshop [119], and Sotozono et al.’s grading system could provide comprehensive assessment of OSD and a framework for more consistent descriptions [120]. Since EB, AIBD, and SJS consist of comparable cicatrising complications, these studies could serve as model platforms to quantify clinical signs for stratification into ‘activity’ or ‘damage’.

Furthermore, the OSD Scoring System Study Group developed an international Delphi consultation in 2017, which highlighted the importance of categorising ocular manifestations [121]. ‘Activity’ would represent findings resulting from inflammatory processes that can be reversed with time or interventions, while ‘damage’ is persistent (≥6 months) and results in permanent changes to anatomy, physiology, pathology, or function. This distinct classification of parameters improves on tools mentioned in this review by providing a standardised, objective tracking of disease severity and progression [122]. The authors also noted the benefits of creating consistent terminology that could be used in a robust system to score outcome comparisons for future clinical trials and evaluation of therapeutic interventions. Furthermore, the article lists the incongruences of current OSD scoring systems and aimed to rectify this by creating the first universal ‘toolbox’ of parameters agreed upon by a panel of international ophthalmic specialists.

A review of the ophthalmic and dermatological literature reveals that scoring systems are beginning to make distinctions between disease ‘activity’ and ‘damage’ [91, 105, 113, 115]. This delineation between ‘activity’ and ‘damage’ could provide a foundation for the future development of a validated and comprehensive EB/AIBD ocular involvement scoring system.

## Conclusion

A review of the literature shows that ocular involvement in EB/AIBD is well documented. Although manifestations can vary in prevalence and severity between the different EB/AIBD subtypes, they are predominantly OSD complications and located to the anterior segment of the eye. The review also highlights that existing scoring tools exhibit considerable shortcomings. To the best of our knowledge, there is no recognised or validated system that comprehensively stages and scores the spectrum of ocular complications in EB/AIBD. A validated system is crucial for objective evaluation of intervention outcomes, longitudinal monitoring, and formulation of treatment plans. Since EB/AIBD patients inherently require multidisciplinary care, a validated ocular involvement scoring system could provide much needed commonality among clinicians, and objectivity for accurate assessment of disease severity and progression.

## Abbreviations

AIBD: Autoimmune blistering disease
BMZ: Basement membrane zone
BP: Bullous pemphigoid
BP180: Bullous pemphigoid antigen 2
BP230: Bullous pemphigoid antigen 1
DC: Dendritic cell
DDEB: Dominant dystrophic epidermolysis bullosa
DEB: Dystrophic epidermolysis bullosa
DED: Dry eye disease
EB: Epidermolysis bullosa
EBA: Epidermolysis bullosa acquisita
EBDASI: Epidermolysis bullosa disease activity and scarring index
EBS: Epidermolysis bullosa simplex
IVCM: In vivo confocal microscopy
JEB: Junctional epidermolysis bullosa
KS: Kindler syndrome
LABD: Linear immunoglobulin-A bullous dermatosis
MMP: Mucous membrane pemphigoid
NEBR: National EB Registry
OcMMP: Mucous membrane pemphigoid with ocular involvement
OSD: Ocular surface disease
PF: Pemphigus foliaceus
PNP: Paraneoplastic pemphigus
PV: Pemphigus vulgaris
RDEB: Recessive dystrophic epidermolysis bullosa
